# Prevalence of and factors associated with sarcopenia in patients on hemodialysis in Brazil: findings from the multicenter SARC-HD study

**DOI:** 10.3389/fmed.2025.1671237

**Published:** 2026-01-05

**Authors:** Marvery P. Duarte, Otávio T. Nóbrega, Barbara P. Vogt, Marina S. Pereira, Fábio A. Vieira, Maryanne Z. C. Silva, Henrique S. Disessa, Dario R. Mondini, Rodrigo R. Krug, Bruna M. Sant’Helena, Daiana C. Bundchen, Maristela Bohlke, Angélica N. Adamoli, Ricardo M. Lima, Antônio J. Inda-Filho, Carla M. Avesani, Maycon M. Reboredo, Heitor S. Ribeiro, the SARC-HD Study Group

**Affiliations:** 1 Faculty of Health Sciences, University of Brasilia, Brasília, Brazil; 2 Medicine Faculty, Federal University of Uberlandia, Uberlândia, Brazil; 3 School of Medicine, Federal University of Juiz de Fora, Juiz de Fora, Brazil; 4 Internal Medicine Department, Botucatu Medical School, São Paulo State University, UNESP, Botucatu, Brazil; 5 Department of Physical Education, São Paulo State University, Bauru, Brazil; 6 Laboratory of Applied Kinesiology, Faculty of Physical Education, State University of Campinas, Campinas, Brazil; 7 Postgraduation Program in Comprehensive Health Care, University of Cruz Alta, Cruz Alta, Brazil; 8 IELUSC Faculty, Joinville, Brazil; 9 Department of Health Sciences, Federal University of Santa Catarina, Araranguá, Brazil; 10 Postgraduate Program in Health and Behavior, Catholic University of Pelotas, Pelotas, Brazil; 11 Hospital de Clínicas de Porto Alegre, Porto Alegre, Brazil; 12 Faculty of Physical Education, University of Brasilia, Brasília, Brazil; 13 Department of Clinical Science, Technology and Intervention, Division of Renal Medicine and Baxter Novum, Karolinska Institute, Stockholm, Sweden

**Keywords:** chronic kidney disease, dialysis, handgrip strength, calf circumference, epidemiology, multicenter study

## Abstract

**Objective:**

The understanding of sarcopenia in patients on hemodialysis from middle-income countries remains underexplored. We investigated the prevalence of and factors associated with sarcopenia in patients undergoing hemodialysis in Brazil.

**Methods:**

This was a cross-sectional analysis using baseline data from the ***SARC**openia trajectories and associations with clinical outcomes in patients on **H**emo**D**ialysis* (SARC-HD) multicenter study. Muscle strength was assessed by handgrip, muscle mass by calf circumference, and physical performance by the 4-m gait speed test. Sarcopenia was diagnosed and staged as probable, confirmed, or severe based on the revised European Working Group on Sarcopenia in Older People (EWGSOP2) consensus. Associated factors were investigated with adjustment for potential confounders.

**Results:**

A total of 983 patients (median age 59 years; 48% ≥ 60 years; 40% female) from 19 dialysis centers were analyzed. The prevalences of probable, confirmed, and severe sarcopenia were 12, 9, and 5%, respectively. Sarcopenia prevalence increased with age, ranging from 7 to 45% in the male individuals and from 4 to 21% in the female individuals. In the fully adjusted model, older age [≥60 years; adjusted odds ratio (aOR): 3.30, 95% confidence interval (CI): 2.09–5.21], male sex (aOR: 1.77, 95% CI: 1.13–2.77), white ethnicity (aOR: 1.88, 95% CI: 1.23–2.87), and diabetes as the etiology/comorbidity of chronic kidney disease (CKD) (aOR: 1.83, 95% CI: 1.20–2.91) were independently associated with higher odds of sarcopenia. Nevertheless, overweight (aOR: 0.38, 95% CI: 0.24–0.60) and obesity (aOR: 0.11, 95% CI: 0.05–0.26) were associated with lower odds. After sensitivity analyses based on dialysis prescription, only older age, white ethnicity, overweight, and obesity remained independently associated with sarcopenia.

**Conclusion:**

One in four patients on hemodialysis had some stage of sarcopenia. Independent risk factors associated with sarcopenia were older age and white ethnicity, whereas overweight and obesity were protective factors. These findings may help identify hemodialysis patients at increased risk of sarcopenia, thereby promoting early screening, diagnosis, and treatment strategies.

## 1 Introduction

Sarcopenia is an age-related condition characterized by low levels of physical function and skeletal muscle mass (1, 2). In patients with chronic diseases, such as chronic kidney disease (CKD), an accelerated aging process may predispose them to a more rapid decline in skeletal muscle health, mainly due to low-grade chronic inflammation, oxidative stress, malnutrition, and immunosenescence (3, 4). This accelerated aging process promotes muscle wasting (5, 6), leading to an earlier onset of sarcopenia traits. Previous systematic reviews have shown that the prevalence of sarcopenia in chronic diseases is heterogeneous: 18% in diabetes (7), 16% in chronic obstructive pulmonary disease (8), 38% in cirrhosis (9), and 22% during cancer treatment (10). In patients with CKD at all stages, the overall prevalence of sarcopenia is 25%, while it is 29% among those on hemodialysis (11).

Screening for and diagnosing sarcopenia in chronic diseases, such as kidney failure requiring renal replacement therapy—which is highly prevalent in Brazil (12)—is essential to identify patients at higher risk of adverse clinical outcomes. Patients on dialysis with sarcopenia may have a twofold increased risk of mortality (13, 14). In the general population, malnutrition, smoking, extreme sleep duration, and diabetes have been recently identified as significant factors associated with sarcopenia (15). Furthermore, modifiable lifestyle factors, particularly physical inactivity, are recognized as significant contributors to muscle wasting and physical function decline in chronic diseases, including CKD (15). Data on the prevalence of sarcopenia and its associated factors in patients on hemodialysis are largely derived from single-center studies (11), with little evidence from large multicenter studies. To the best of our knowledge, no large-scale multicenter study has been conducted in Latin American countries to date.

To address the knowledge gap regarding the epidemiological profile of sarcopenia in patients on hemodialysis in middle-income countries, especially Latin America, we investigated its prevalence and associated factors in a multicenter sample from different regions of Brazil.

## 2 Methods

### 2.1 Design, setting, and population

This cross-sectional report is part of the ***SARC**openia trajectories and associations with clinical outcomes in patients on **H**emo**D**ialysis* (**SARC-HD**) study, a large national multicenter prospective cohort that has been conducted at 19 dialysis centers across Brazil since October 2022. Recruitment and baseline assessments occurred between October 2022 and April 2023. A more detailed description of the design and methodology has been published elsewhere (16). Briefly, patients aged 18 years or older undergoing maintenance hemodialysis for at least 3 months were eligible for participation. The exclusion criteria included physical limitations, limb amputations, and other conditions or medical contraindications that would prevent any physical function assessment. All participants provided written informed consent, and the Institutional Review Board of the University Center ICESP (no. 5.418.365) ethically approved the study (all other institutional review boards reviewed and agreed with the approval letter). This study adhered to the principles of the Declaration of Helsinki. The SARC-HD study is also registered at the *Registro Brasileiro de Ensaios Clínicos* (ReBEC) platform (RBR-82p87rq). This manuscript was written in accordance with the Strengthening the Reporting of Observational Studies in Epidemiology (STROBE) Statement.

### 2.2 Sociodemographic and clinical variables

Clinical (e.g., comorbidities, CKD etiology) and demographic data were collected from electronic health records by the same experienced researcher at each dialysis center. Missing information was obtained from patients or the medical team. Patients aged ≥ 60 years were considered older according to the Brazilian Statute of the Elderly. Ethnicity was self-reported according to the Brazilian Institute of Geography and Statistics as white, black, brown (*Pardo*, a mixed black and white ethnicity), or other (East Asian, Indigenous, or Quilombola). For further analysis, we stratified the patients into *white* (East Asian and white) and *non-white* (black, brown, Indigenous, and Quilombola) groups due to the characteristics of miscegenation in the Brazilian population. Brazilian minimum wages (based on 2022; 1,212 BRL or approximately 220 USD) were self-reported according to the patients’ individual income, not their household income. Smoking and alcohol consumption were reported as *Yes* (current), *No* (former), or *Never*. The patients were stratified according to the weekly frequency of dialysis as conventional (3 sessions) or short daily (≥ 4 sessions). Body mass index (BMI; kg/m^2^) was calculated and classified according to the World Health Organization (WHO) criteria: overweight, 25.0–29.9 kg/m^2^, and obesity, ≥30.0 kg/m^2^. Physical activity was assessed using the short version of the International Physical Activity Questionnaire (IPAQ), and the patients were classified as physically active (≥ 150 min/week) or inactive (< 150 min/week), in accordance with the WHO recommendations (17).

### 2.3 Laboratory parameters

Blood samples were collected before the first weekly dialysis session, following operational procedures recommended by the Brazilian Ministry of Health (18). Assays were conducted in affiliated laboratories at each dialysis center. Serum levels of sodium, potassium, phosphorus, calcium, albumin, and intact parathyroid hormone (iPTH) were assessed. Cutoff values for electrolyte imbalances varied depending on the laboratory; therefore, we standardized them using the KDIGO (19) or National Kidney Foundation (20) recommendations, unless otherwise specified. A detailed description is provided in Supplementary Table 1.

### 2.4 Assessment of sarcopenia parameters

Measurements of physical function (i.e., muscle strength and physical performance) were conducted before a midweek dialysis session by an experienced researcher at each dialysis center. Detailed descriptions of the protocols have been published previously (16).

#### 2.4.1 Muscle strength

##### 2.4.1.1 Handgrip strength

Maximal voluntary isometric contraction was assessed using one of two hydraulic dynamometers, the Jamar (Sammons Preston Rolyan, Bolingbrook, IL, United States) or the Saehan (Saehan Corp., Changwon, Korea), depending on availability at each dialysis center. These two dynamometers present an excellent intraclass correlation coefficient (21). The highest value obtained from three trials in each arm was recorded and reported in kilograms (kg) (22).

##### 2.4.1.2 Five-time sit-to-stand test

Lower-limb muscle strength was assessed using the five-time sit-to-stand (STS-5) test. The shortest duration, in seconds, from three trials was recorded (23).

#### 2.4.2 Physical performance

##### 2.4.2.1 Gait speed

Physical performance was assessed by measuring usual gait speed over four meters. A total of three attempts were made, and the distance divided by the shortest time [i.e., speed (m/s)] was used for analysis (24).

#### 2.4.3 Calf circumference

Calf circumference was assessed using a non-stretchable measuring tape while patients were seated without muscle contraction, at the point of greatest circumference on the right leg (25). The mean of two measurements was used for analysis and reported in centimeters. If a patient presented with clinical signs of edema during the nephrologist’s evaluation, the assessment was postponed and conducted in a subsequent session with adequate volume status. Measurements were taken after a midweek dialysis session.

### 2.5 Diagnosis of sarcopenia

Sarcopenia diagnosis and staging were based on the revised European Working Group on Sarcopenia in Older People (EWGSOP2) criteria, classified as no sarcopenia, probable sarcopenia, confirmed sarcopenia, and severe sarcopenia (26). Probable sarcopenia was defined as low muscle strength alone (handgrip strength < 27 kg for male individuals and < 16 kg for female individuals). Confirmed sarcopenia was defined as low muscle strength accompanied by low muscle mass (calf circumference ≤34 cm for male individuals and ≤33 cm for female individuals) (27). Severe sarcopenia was defined as a diagnosis of confirmed sarcopenia *plus* low physical performance (gait speed ≤ 0.8 m/s for both sexes). We grouped confirmed and severe sarcopenia as ‘sarcopenia’ for further analysis. In the case of missing data for calf circumference or gait speed, only the probable sarcopenia stage was assessed.

### 2.6 Statistical analysis

#### 2.6.1 Sample size

A sample size calculation was not performed, as we aimed to recruit as many eligible patients as possible from the cohort population.

#### 2.6.2 Missing data and imputation

Details of missing data are provided in Supplementary Table 2. For sarcopenia-related variables, no data imputation was conducted. However, for descriptive continuous variables (<15% missing data), we applied the multiple imputation regression method, assuming the data were missing at random (28). Age, BMI, and sex were used as predictors, whereas the variables of imputation interest were not included.

#### 2.6.3 Descriptive analysis

Data normality was assessed through visual inspection of histograms and the Kolmogorov–Smirnov test. Continuous data were presented as median and interquartile range (IQR), whereas categorical data were presented as frequencies and valid percentages. Comparisons among the groups based on sarcopenia stages were conducted using the Kruskal–Wallis test with Bonferroni post-hoc correction for continuous variables and the chi-squared or Fisher’s exact test for categorical variables.

#### 2.6.4 Binary logistic regression

Binary logistic regression analyses were conducted to identify factors independently associated with the presence of sarcopenia (confirmed *plus* severe sarcopenia). Odds ratios (ORs) and 95% confidence intervals (CIs) were calculated. Clinical and dialysis prescription variables with a *p-*value of <0.25 in the between-group comparison (no sarcopenia *vs.* sarcopenia) were included in the models for adjustments. A total of two models were developed using both stepwise and backward selection approaches. Variables were retained in the model for adjustment if their exclusion led to a change of more than 10% in the parameter estimates of the remaining variables. Model goodness-of-fit was assessed using the Hosmer–Lemeshow test, and the significance of each variable was determined using the Wald test.

Model 1 (n = 943) included clinical variables: age (reference = <60 years), ethnicity (reference = non-white), sex (reference = female), diabetes as the etiology or comorbidity of CKD (reference = no diabetes), body mass index (reference = normal weight), and physically active (reference = <150 min/week). Model 2 (n = 945) included all variables from model 1, with the addition of dialysis modality (reference = conventional) and vascular access (reference = arteriovenous fistula or graft). Sensitivity analyses were conducted by excluding patients undergoing hemodiafiltration or receiving short daily dialysis (≥ 4 sessions/week).

Analyses were performed using Statistical Package for the Social Sciences (version 29.0, SPSS Inc., Chicago, United States) and GraphPad Prism (version 8.4, GraphPad Software, San Diego, USA). A two-tailed *p-*value of <0.05 was considered statistically significant.

## 3 Results

### 3.1 Recruitment

From the 19 dialysis centers included in the SARC-HD study, a total of 1,525 patients were assessed for eligibility; 1,008 were recruited, and 983 were included in the final analysis (see Supplementary Figure 1 for the flowchart). A detailed description of recruitment in each city is provided in Supplementary Figure 2; most of the patients were from the Federal District (*n* = 485; 49%) and São Paulo state (*n* = 168; 17%). A total of three Brazilian regions were included: 485 patients from the Midwest (49%), 304 from the Southwest (31%), and 194 from the South (20%). Of the dialysis centers included, nine (47.4%) were funded solely by private health insurance companies, three (15.8%) received only public funding from the Brazilian Unified Health System, and seven (36.8%) had mixed funding (private and public).

### 3.2 Characteristics of the patients

The prevalences of probable, confirmed, and severe sarcopenia were 12, 9, and 5%, respectively (Supplementary Figure 3). Individually, low muscle strength, low muscle mass, and low physical performance were found in 26% (*n* = 254), 43% (*n* = 391), and 17% (*n* = 158) of the sample, respectively. The characterization of the cohort is presented in Table 1 and Supplementary Table 3. Male individuals were more frequent in the sarcopenia groups (*p* < 0.001). The more advanced the sarcopenia stage, the older the age (*p* < 0.001) and the higher the frequency of individuals aged ≥ 60 years (*p* < 0.001). Ethnicity differed significantly among the groups, with a higher frequency of white patients in the sarcopenia groups (*p* < 0.001). Marital status, education level, minimum wages, and smoking or alcohol consumption did not differ among the groups (all *p* > 0.05).

**Table 1:** Characteristics of the patients on hemodialysis according to sarcopenia status.

Variables	All patients	No sarcopenia	Probable sarcopenia	Confirmed sarcopenia	Severe sarcopenia	*p*-value
*n* (%)	983 (100)	729 (74.2)	115 (11.7)	88 (9.0)	51 (5.2)	
Male	595 (60.5)	414 (56.8)	81 (70.4)	66 (75.0)	34 (66.7)	0.003
Age (years)	59 [47–69]	56 [44–66]	65 [56–74]^a^	66 [57–72]^a^	72 [68–80]^a,b,c^	< 0.001
≥ 60 years, *n* (%)	475 (48.3)	293 (40.2)	77 (67.0)	59 (67.0)	46 (90.2)	< 0.001
Ethnicity, *n* (%)						< 0.001
White	497 (50.6)	336 (46.1)	74 (64.3)	52 (59.1)	35 (70.0)	
Non-white	485 (49.4)	393 (53.9)	41 (35.7)	36 (40.9)	15 (30.0)	
Comorbidities, *n* (%)						
Diabetes	407 (41.5)	269 (36.9)	60 (50.2)	47 (53.4)	31 (63.3)	< 0.001
Hypertension	814 (83.0)	603 (82.8)	97 (84.3)	72 (81.8)	42 (84.0)	0.963
Cancer	48 (4.9)	30 (4.1)	8 (7.0)	6 (6.9)	4 (8.0)	0.186
COPD	32 (3.3)	16 (2.2)	7 (6.1)	6 (6.9)	3 (6.0)	0.008
Heart failure	152 (15.5)	93 (12.8)	31 (27.0)	13 (14.9)	15 (30.0)	< 0.001
CAD	174 (17.8)	109 (15.0)	27 (23.5)	23 (26.4)	15 (30.0)	< 0.001
Neuropathies	77 (7.8)	52 (7.2)	11 (9.6)	7 (8.0)	7 (14.0)	0.314
No. of comorbidities	2 [1–3]	2 [1–3]	3 [2–4]^a^	2 [2–3]^a^	3 [2–4]^a^	< 0.001
Diabetes as CKD etiology	220 (22.7)	144 (20.1)	30 (26.3)	30 (34.1)	16 (32.0)	0.005
Electrolyte imbalances, *n* (%)						
Hyponatremia (< 135.0 mEq/L)	101 (10.3)	76 (10.4)	11 (9.6)	7 (8.0)	7 (13.7)	0.740
Hyperkalemia (> 5.0 mEq/L)	486 (49.4)	374 (51.3)	48 (41.7)	40 (45.5)	24 (47.1)	0.220
Hyperphosphatemia (> 4.5 mg/dL)	629 (64.0)	482 (66.1)	70 (60.9)	57 (64.8)	20 (39.2)	0.001
Hypocalcemia (< 8.5 mg/dL)	321 (32.7)	235 (32.2)	43 (37.4)	29 (33.0)	14 (27.5)	0.601
Hypoalbuminemia (< 3.5 g/dL)	104 (10.6)	70 (9.6)	14 (12.2)	9 (10.2)	11 (21.6)	0.056
Low iPTH (< 150 pg./mL)	199 (20.2)	142 (19.5)	21 (18.3)	20 (22.7)	16 (31.4)	0.039
High iPTH (> 600 pg./mL)	270 (27.5)	218 (29.9)	24 (20.9)	21 (23.9)	7 (13.7)	
Dialysis modality, *n* (%)						< 0.001
Conventional	676 (68.8)	520 (71.3)	76 (66.7)	61 (69.3)	19 (37.3)	
Hemodiafiltration	306 (31.2)	209 (28.7)	38 (33.3)	27 (30.7)	32 (62.7)	
Dialysis vintage (months)	33 [14–66]	34 [16–63]	26 [10–71]	29 [9–65]	40 [14–83]	0.168
Weekly frequency, *n* (%)						
Conventional (3 sessions)	695 (70.7)	518 (71.1)	86 (74.8)	63 (71.6)	28 (54.9)	0.067
Short daily (≥ 4 sessions)	288 (29.3)	211 (28.9)	29 (25.2)	25 (28.4)	23 (45.1)	
Vascular access, *n* (%)						< 0.001
Arteriovenous fistula	673 (68.5)	526 (72.2)	69 (60.0)	53 (60.2)	25 (49.0)	
Catheter	246 (25.0)	152 (20.9)	42 (36.5)	29 (33.0)	23 (45.1)	
Graft	64 (6.5)	51 (7.0)	4 (3.5)	6 (6.8)	3 (5.9)	
Body composition						
Body mass index (kg/m^2^)*	25.0 [22.3–28.7]	25.3 [22.6–28.9]	26.8 [23.4–30.7]^a^	22.9 [19.9–26.1]^a,b^	23.3 [20.8–24.4]^a,b^	< 0.001
Underweight, n (%)	7 (0.7)	5 (0.7)	0 (0)	2 (2.4)	0 (0)	< 0.001
Normal weight, n (%)	445 (46.9)	315 (44.9)	40 (34.8)	54 (65.9)	36 (73.5)	
Overweight, n (%)	318 (33.5)	244 (34.8)	41 (35.7)	23 (28.0)	10 (20.4)	
Obese, n (%)	178 (18.8)	138 (19.7)	34 (29.6)	3 (3.7)	3 (6.1)	
Calf circumference (cm)	34.5 [32.0–37.0]	35.5 [32.5–37.5]	36.0 [35.0–38.0]^a^	29.2 [31.3–33.0]^a,b^	29.0 [28.2–31.5]^a,b^	< 0.001
Female	33.5 [31.5–36.5]	35.1 [32.0–37.0]	35.1 [34.0–37.0]^a^	30.5 [27.4–32.4]^a,b^	29.0 [27.4–31.3]^a,b^	< 0.001
Male	35.0 [32.5–37.1]	35.5 [33.5–38.0]	36.5 [35.0–38.0]^a^	31.5 [30.0–33.1]^a,b^	30.0 [29.0–31.7]^a,b^	< 0.001
Physical function						
Handgrip strength (kg)	26 [20–34]	30 [24–38]	18 [14–24]^a^	20 [14–24]^a^	14 [13–20]^a^	< 0.001
Female	22 [17–26]	22 [20–26]	12 [11–14]^a^	13 [11–14]^a^	13 [11–14]^a^	< 0.001
Male	32 [25–39]	36 [30–40]	22 [18–25]^a^	22 [18–24]^a^	18 [14–22]^a^	< 0.001
Gait speed (m/s)	1.10 [0.91–1.30]	1.14 [0.98–1.33]	1.00 [0.74–1.17]^a^	1.09 [0.96–1.21]	0.60 [0.39–0.74]^a,b,c^	< 0.001
Female	1.05 [0.86–1.24]	1.08 [0.91–1.27]	0.87 [0.65–1.05]^a^	1.02 [0.90–1.15]	0.73 [0.28–0.77]^a,b,c^	< 0.001
Male	1.14 [0.95–1.33]	1.18 [1.03–1.38]	1.05 [0.82–1.20]^a^	1.10 [0.97–1.22]	0.60 [0.40–0.69]^a,b,c^	< 0.001
Five-time sit-to-stand (seconds)	12.2 [9.8–15.4]	11.7 [9.4–14.8]	13.7 [11.0–17.1]^a^	13.5 [11.1–16.1]^a^	16.3 [13.1–22.2]^a^	< 0.001
Female	12.4 [10.1–16.0]	12.1 [9.9–15.3]	13.1 [10.3–17.7]^a^	13.1 [11.4–17.5]^a^	16.3 [11.6–24.6]^a^	< 0.001
Male	12.1 [9.5–15.1]	11.5 [9.0–14.4]	13.7 [11.2–17.0]^a^	13.5 [10.9–16.0]^a^	16.9 [13.1–21.0]^a^	< 0.001
Physically active, *n* (%)	258 (26.2)	213 (29.2)	25 (21.7)	14 (15.9)	6 (11.8)	0.002

CAD, coronary artery disease; COPD, chronic obstructive pulmonary disease; CKD, chronic kidney disease; iPTH, intact parathyroid hormone. *For the classification of body mass index, imputed data were not considered. ^a–c^Indicate significant differences compared to the no sarcopenia, probable sarcopenia, and confirmed sarcopenia groups, respectively.

The sarcopenia groups showed a higher frequency of comorbidities, such as diabetes (*p* < 0.001), chronic obstructive pulmonary disease (*p* = 0.008), heart failure (*p* < 0.001), and coronary artery disease (*p* < 0.001). Diabetes as the etiology of CKD was also more frequent in the sarcopenia groups (*p* < 0.001). In the severe sarcopenia group, the frequency of hyperphosphatemia was lower (*p* = 0.001), whereas low and high iPTH values were more and less frequent, respectively (31.4 and 13.7%, *p* = 0.039). There were significant differences among the groups in terms of dialysis modality (*p* < 0.001), dialysis weekly frequency (*p* = 0.007), and vascular access (*p* < 0.001), whereas dialysis vintage did not differ (*p* = 0.168).

The body composition and physical function variables of the patients according to sarcopenia stages are provided in Table 1. The patients with sarcopenia (confirmed and severe) had lower BMI (*p* < 0.001) than those without sarcopenia. Compared to the confirmed sarcopenia group, the patients with severe sarcopenia only differed in gait speed, showing lower performance (*p* < 0.001). Supplementary Figure 4 shows the linear association of physical function and calf circumference with advancing age. The patients with sarcopenia were less physically active (*p* = 0.002).

### 3.3 Prevalence of sarcopenia among the subgroups

**Figure 1:**
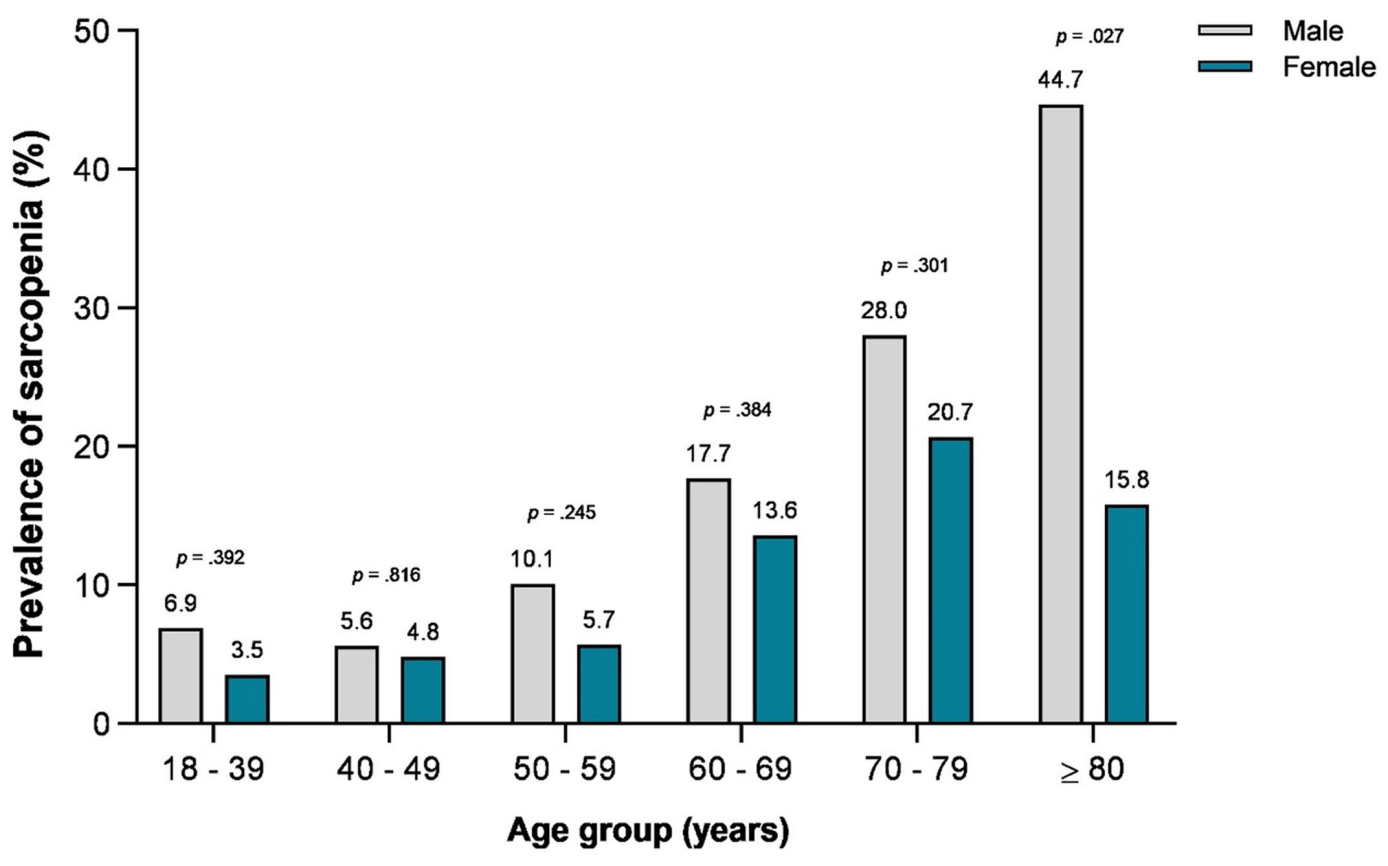
Prevalence of sarcopenia stratified by age group and sex.

The prevalence of sarcopenia increased with advancing age, ranging from 7 to 45% in the male patients and from 4 to 21% in the female patients (Figure 1). The male patients had a higher prevalence of sarcopenia in the oldest age group (≥ 80 years; 45 *vs.* 16%; *p* = 0.027).

**Figure 2:**
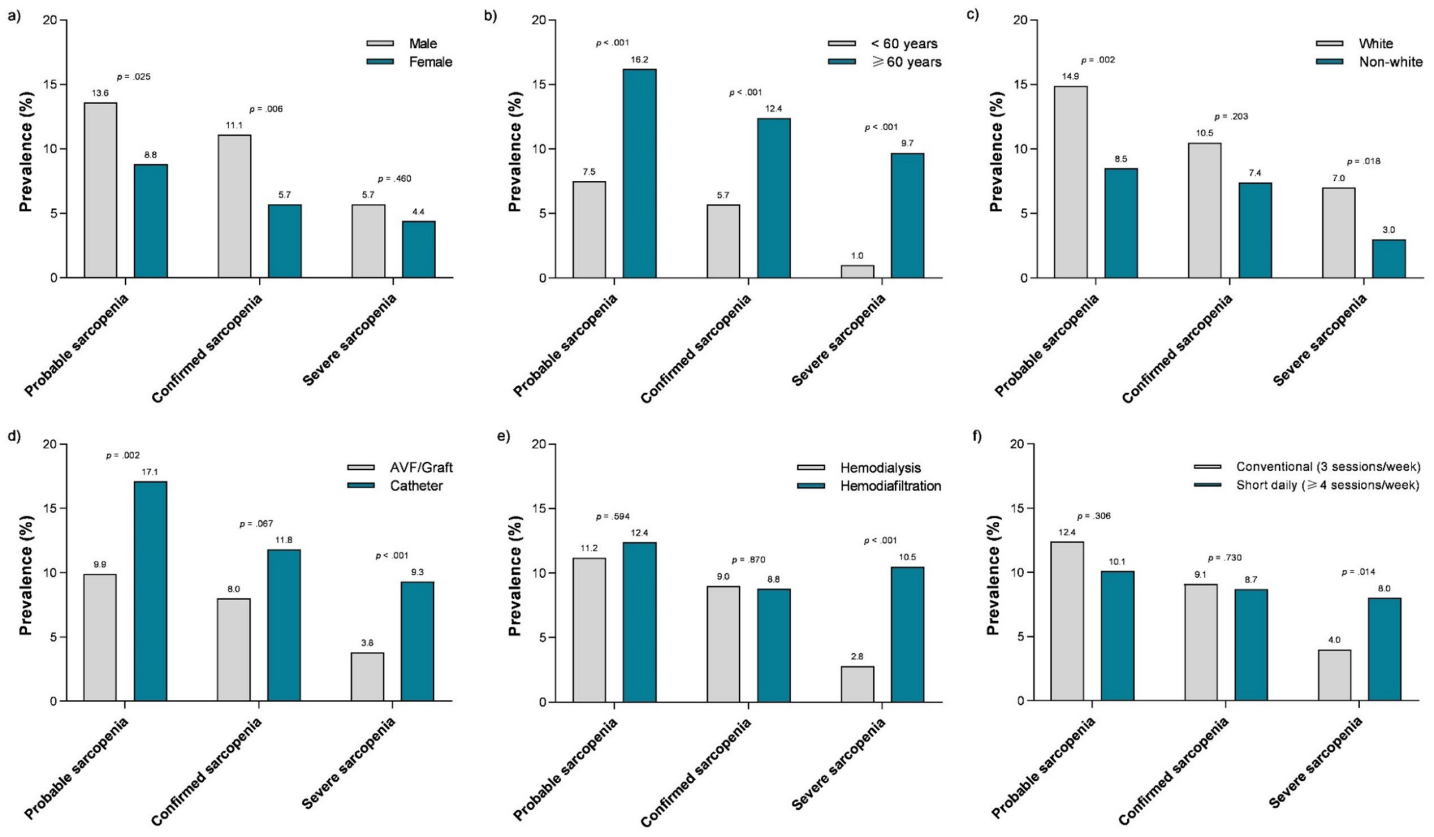
Prevalence of sarcopenia stages according to sex **(A)**, age **(B)**, ethnicity **(C)**, vascular access **(D)**, dialysis modality **(E)**, and weekly dialysis frequency **(F)**. AVF, arteriovenous fistula.

Regarding sarcopenia stages, Figure 2 shows that the male patients (panel a) had a higher prevalence of probable (14 *vs*. 9%; *p* = 0.025) and confirmed sarcopenia (11 *vs*. 6%; *p* = 0.006). Probable, confirmed, and severe sarcopenia were more prevalent among the older patients (≥ 60 years; all *p* < 0.001; panel b). The patients of white ethnicity (panel c) had a higher prevalence of probable (15 *vs*. 9%; *p* = 0.002) and severe sarcopenia (7 vs. 3%; *p* = 0.018). Probable (17 *vs*. 10%; *p* = 0.002) and severe sarcopenia (9 vs. 4%; *p* < 0.001) were more prevalent among the patients with a catheter as vascular access (panel d). Regarding dialysis characteristics, the patients undergoing hemodiafiltration (panel e) and on a short daily frequency (≥ 4 sessions; panel f) had higher rates of severe sarcopenia (11 *vs*. 3%; *p* < 0.001; and 8 *vs*. 4%; *p* = 0.014, respectively).

### 3.4 Factors associated with sarcopenia

Table 2 describes the factors associated with sarcopenia. In the fully adjusted model 2, older age (aOR: 3.03, 95% CI: 1.90–4.82), male sex (aOR: 1.77, 95% CI: 1.13–2.78), white ethnicity (aOR: 1.92, 95% CI: 1.26–2.94), and diabetes as the etiology or comorbidity of CKD (aOR: 1.78, 95% CI: 1.16–2.73) were independently associated with higher odds of sarcopenia. On the other hand, overweight (aOR: 0.37, 95% CI: 0.23–0.59) and obesity (aOR: 0.11, 95% CI: 0.05–0.26) were independently associated with lower odds of sarcopenia.

**Table 2:** Identification of factors associated with sarcopenia in the patients on hemodialysis.

Variables	Adjusted model 1		Adjusted model 2	
	Odds ratio (95% CI)	*p*-value	Odds ratio (95% CI)	*p*-value
Older age (≥ 60 years)	3.14 (1.98–4.98)	<0.001	3.03 (1.90–4.82)	<0.001
Male sex	1.70 (1.09–2.65)	0.019	1.77 (1.13–2.78)	0.013
White ethnicity	2.02 (1.33–3.08)	0.001	1.92 (1.26–2.94)	0.003
Diabetes as CKD etiology or comorbidity	1.96 (1.29–2.98)	0.002	1.78 (1.16–2.73)	0.008
Body mass index				
Normal weight	Reference			
Underweight	4.38 (0.67–28.55)	0.122	3.99 (0.57–28.01)	0.164
Overweight	0.36 (0.23–0.57)	<0.001	0.37 (0.23–0.59)	<0.001
Obesity	0.10 (0.04–0.25)	<0.001	0.11 (0.05–0.26)	<0.001
Hemodiafiltration (reference = conventional)	–		1.45 (0.95–2.21)	0.085
Catheter access (reference = arteriovenous fistula)	–		1.48 (0.95–2.30)	0.084
Physically active	0.54 (0.31–0.94)	0.028	0.59 (0.33–1.04)	0.066

CKD, chronic kidney disease; CI, confidence interval. Confirmed and severe sarcopenia groups were combined and classified as ‘sarcopenia’ for analysis. Adjusted model 1 included age (reference = <60 years), ethnicity (reference = non-white), sex (reference = female), diabetes as etiology or comorbidity (reference = no diabetes), body mass index (reference = normal weight), and physically active (reference = <150 min/week). Adjusted model 2 included all variables from model 1, with the addition of dialysis modality (reference = conventional) and vascular access (reference = arteriovenous fistula or graft).

### 3.5 Sensitivity analyses of the factors associated with sarcopenia

Sensitivity analyses of the factors independently associated with sarcopenia in the overall sample were conducted (Supplementary Table 4) by excluding the patients undergoing hemodiafiltration and on short daily dialysis frequency (≥ 4 sessions/week). The results were partially consistent with the overall analysis, confirming that older age (≥ 60 years), white ethnicity, overweight, and obesity were independently associated with sarcopenia. Conversely, male sex and diabetes as the etiology or comorbidity of CKD showed different patterns of association depending on the sensitivity analysis.

## 4 Discussion

### 4.1 Main findings

In this *multicenter study* in Brazil, we examined the prevalence of and factors associated with sarcopenia in patients on hemodialysis. Our findings indicated that the prevalences of probable, confirmed, and severe sarcopenia stages were 12, 9, and 5%, respectively. Overall, some stage of sarcopenia was observed in 25% of the patients. The patients with sarcopenia (i.e., confirmed + severe) were mainly male, older, and of white ethnicity; had more diabetes; received hemodiafiltration treatment more frequently; used a catheter as vascular access; were less physically active; and had lower BMI. After multiple sensitivity analyses based on clinical and dialysis characteristics, key risk factors associated with sarcopenia included older age and white ethnicity, whereas overweight and obesity were associated with lower odds. These findings, which enhance our understanding of sarcopenia in patients on hemodialysis in Brazil, may help health professionals and researchers identify patients at increased risk of sarcopenia, thereby promoting early screening, diagnosis, and treatment strategies.

### 4.2 Overall prevalence of sarcopenia

Previous evidence on the prevalence of sarcopenia in patients with kidney failure receiving replacement therapy has been mainly from single-center studies and did not include a wide range of dialysis regimens, as in the present sample. Sarcopenia was diagnosed in 14% of our sample. A Chinese multicenter study by Zhou et al. included 3,196 patients undergoing maintenance hemodialysis from 20 centers and reported a sarcopenia prevalence of 36% using the Asian Working Group for Sarcopenia (AWGS) definition (29). This difference between Zhou et al. and our findings may be due to the different consensus criteria used. Kittiskulnam et al. (30), using data from the ACTIVE/ADIPOSE studies conducted at 14 hemodialysis centers in the United States of America and including 645 patients, reported a sarcopenia prevalence ranging from 4 to 16%, depending on the cutoff for low muscle mass according to the EWGSOP consensus (31). Individually, low muscle mass/height squared, low handgrip strength, and low gait speed (i.e., sarcopenia traits) were identified in 8, 30, and 35% of patients, respectively, in the ACTIVE/ADIPOSE studies. The same sarcopenia traits were found in 43, 26, and 17% of the patients in our Brazilian sample. Zhou et al. (29), however, did not evaluate these traits individually.

The application of different consensus criteria may impact the prevalence of sarcopenia (11). Kittiskulnam et al. (30) applied the same consensus that we employed, but they used a former definition (31). Despite finding a similar prevalence of sarcopenia, they found differences in sarcopenia traits, especially in low muscle mass. We assessed muscle mass by measuring calf circumference, whereas Kittiskulnam et al. used bioelectrical impedance spectroscopy (BIS), which may explain the heterogeneity. Despite being different methods to estimate muscle mass, previous studies in patients on hemodialysis have shown a strong and significant correlation between BIS-derived skeletal muscle mass and calf circumference (32). This emphasizes that when a BIS assessment is not available, calf circumference could be used as a marker of muscle mass.

Altogether, until there is an internationally accepted operational diagnosis of sarcopenia, differences may interfere with clinical practice (33–35). Therefore, the present data should be interpreted with caution based on the cutoff points adopted to determine low muscle strength, low muscle mass, and low physical performance.

### 4.3 Differences in prevalence and factors associated with sarcopenia

Our findings showed that older age, white ethnicity, overweight, and obesity were significantly associated with sarcopenia in the fully adjusted model and sensitivity analyses. There is a plethora of evidence confirming that sarcopenia is mainly an age-related disease (36), and CKD *per se* may contribute to an accelerated aging process. In our adjusted binary logistic regression, older age was associated with more than a threefold increase in the odds of sarcopenia. Therefore, our findings align with the literature, including previous systematic reviews that showed a positive association between the prevalence of sarcopenia and older age (11).

We also identified white ethnicity as an independent factor associated with sarcopenia after adjustment for confounders (88% higher odds compared to the non-white individuals). Bigman and Ryan, using data from the National Health and Nutrition Examination Survey (NHANES) in older adults (≥ 50 years) without CKD, found that non-white individuals (i.e., black) were less likely to have low appendicular lean mass relative to BMI compared to white individuals (37). In patients on hemodialysis, Yoowannakul et al. investigated the effects of ethnicity on sarcopenia prevalence (38). The prevalence of sarcopenia was 37% among Asian individuals (by the AWGS criterion), 19% among black individuals, and 37% among white individuals (both according to the EWGSOP criterion). These findings corroborate ours and indicate a discrepancy in sarcopenia prevalence among ethnicities, particularly in white individuals, but future research should explore the underlying causes.

Interestingly, our findings demonstrated that the patients with overweight or obesity were less likely to have sarcopenia compared to those with normal weight. Higher BMI and better clinical outcomes have been described as the “obesity paradox” (39), and previous studies in patients on hemodialysis from Brazil have confirmed such a phenomenon for mortality (40). Despite the limitations of assessing nutritional status using BMI, our results indicate that overweight and obesity might also play a role in musculoskeletal health, impacting sarcopenia status. Future studies should focus on the coexistence of obesity and sarcopenia (i.e., sarcopenic obesity) and investigate whether the obesity paradox phenomenon—in the presence of sarcopenia—remains clinically meaningful.

In the overall analysis, male sex and diabetes were found to be significantly associated with sarcopenia, but not independently, as significance was lost in some sensitivity analyses. Previously, we found a negative association between the prevalence of sarcopenia and the percentage of female individuals in the sample (11). This inverse association could be explained by testosterone deficiency, one of the major endocrine disorders in male patients with CKD (41–43). As we did not assess testosterone levels, future studies should investigate whether this association is mainly driven by testosterone deficiency.

Diabetes is one of the main causes of CKD worldwide (19), and insulin resistance has been reported as one of the main factors for muscle mass loss (44). In patients with kidney failure receiving replacement therapy, decreased renal gluconeogenesis may lead to reduced intramuscular glycogen reserves, thereby contributing to the development of sarcopenia (45). Even so, diabetes was not independently associated with sarcopenia when patients on hemodiafiltration or on short daily dialysis were excluded from the analysis, suggesting that this association may be influenced by the dialysis regimen.

### 4.4 Strengths and limitations

We consider the multicenter design to be the main strength of our study, as there is limited multicenter evidence regarding sarcopenia in dialysis centers, especially in middle-income countries such as Brazil. In addition, we recruited dialysis centers from three regions and five states in Brazil, encompassing different dialysis regimens, socioeconomic classifications, and ethnicities, which may partially represent the Brazilian population on hemodialysis.

Despite these strengths, we acknowledge that our study has several limitations. (i) Possible heterogeneity in data collection, as the dialysis centers used different electronic health records, routines, and procedures. To mitigate this limitation, we designed standardized tutorials and materials and conducted monthly virtual meetings. (ii) The assessment of calf circumference as a marker of muscle mass, as recommended by the updated EWGSOP2 guideline (26). Despite being an indirect marker, it has been constantly shown to be highly associated with skeletal muscle mass assessed by dual-energy X-ray absorptiometry (46). (iii) The lack of association between physical activity and sarcopenia in the fully adjusted model should be interpreted with caution, as the IPAQ, like other self-reported tools, is subject to recall bias and may not accurately capture the correct volume and intensity of physical activity, particularly in a clinical population with vulnerable health status. Future studies employing objective measures, such as accelerometers, may provide a more precise understanding of the role of being physically active.

Also, (iv) Our sample predominantly consisted of patients undergoing conventional dialysis (i.e., three sessions per week and not on hemodiafiltration); therefore, the findings should be generalized to other dialysis regimens with caution. (v) Excluding participants with physical limitations or amputations may have led to an underestimation of sarcopenia, and the prevalence may be higher than 25%. (vi) Half of our dialysis centers were solely funded by private health insurance companies. Data from the Brazilian Dialysis Registry show that only 20% of Brazilian dialysis centers rely exclusively on this funding source (12). Therefore, caution is warranted when extrapolating the present data to centers exclusively funded by the Brazilian Unified Health System. vii) The cross-sectional design of our study prevents any inference of causality or temporal relationships. Prospective cohort studies are needed to determine whether the variables independently associated with sarcopenia in our study increase the risk of its incidence.

## 5 Conclusion

In conclusion, some stage of sarcopenia was observed in one out of four patients on hemodialysis in our multicenter *SARC-HD study*. The more severe the sarcopenia stage, the lower the prevalence. Clinical characteristics significantly influenced the prevalence of and the association with sarcopenia. We identified older age and white ethnicity as independent risk factors associated with sarcopenia, whereas overweight and obesity were protective factors.

Our findings enhance the understanding of sarcopenia in patients on hemodialysis in Brazil. We advocate that dialysis staff teams in Brazil make concerted efforts to identify patients at increased risk of sarcopenia in their clinical routines by following standard guidelines, such as those recommended by the EWGSOP2, until a national guideline is established.

## Data Availability

The raw data supporting the conclusions of this article will be made available by the authors, without undue reservation.

## Group member of SARC-HD study group

Victor M. Baião, João M. Rocha, João P. Silva, Priscila M. Varela, Jacqueline S. Monteiro, Ana C. Bainha, Emanuele P. Gravina, Abner R. Castro, Fabiana L. Costa, Clara C. Rosa, Henrique L. Monteiro, Luiz R. Medina, Flávio I. Nishimaru, Maria G. Rosa, Ana C. Picinato, Marco C. Uchida, Paulo R. Moreira, Camila R. Vignali, Luane B. de Oliveira, Samy Mendez, Augusto H. Mansur, Catiussa Colling, Aparecido P. Ferreira.
