# Supplementary material for: Prevalence of and factors associated with sarcopenia in patients on hemodialysis in Brazil: findings from the multicenter SARC-HD study
Source: Front Med (Lausanne). 2026 Jan 5;12:1671237. doi: 10.3389/fmed.2025.1671237 (PMC12812607; doi:10.3389/fmed.2025.1671237)
Supplement: Supplementary file 1 [file Supplementary_file_1.docx]

Supplementary Material

**Supplementary Material 1.** Full list of the SARC-HD Study members

**Centers (Federal District)**: 1) DaVita Advance, Brasília, Federal District

2) DaVita Águas Claras, Águas Claras, Federal District

3) DaVita Alvorada, Brasília, Federal District

4) DaVita Ceilândia, Ceilândia, Federal District

5) DaVita Gama, Gama, Federal District

6) DaVita JK, Taguatinga, Federal District

7) DaVita Asa Sul, Brasília, Federal District

8) DaVita Pacini, Brasília, Federal District

**Principal Investigator**: Heitor S. Ribeiro, PhD

**Coordinator**: Marvery P. Duarte, MSc

**Collaborating researchers**: Fábio A. Vieira; Jacqueline Monteiro, MD; Priscila Varela, PT; Victor Baião, MSc; Ricardo M. Lima, PhD; Antônio Inda-Filho, PhD; Aparecido Ferreira, PhD; Otávio T. Nóbrega, PhD.

**Center**: NefroClass, Paulínia, São Paulo

**Principal Investigator**: Marco C. Uchida, PhD

**Coordinator**: Dário R. Mondini

**Collaborating researchers**: Luiz Medina, MS; Luiza Sad, PT; Flávio Nishimaru, MD

**Center**: Renal Quality, Jundiaí, São Paulo

**Principal Investigator**: Marco C. Uchida, PhD

**Coordinator**: Dário R. Mondini

**Collaborating researchers**: Luiz Medina, MSc; Luiza Sad, PT; Maria Gabriela Rosa, MD

**Center**: Unidade de Diálise do Hospital das Clínicas da Faculdade de Medicina de Botucatu (HCFMB), Botucatu, São Paulo

**Principal Investigator**: Maryanne Z. C. Silva, PhD

**Coordinator**: Maryanne Z. C. Silva, PhD

**Collaborating researchers**: Fabiana L. Costa, MSc; Isabele C. Rodrigues; Paula T. Presti; Tabata M. Silva

**Center**: DaVita Bauru, Bauru, São Paulo

**Principal Investigator**: Clara Rosa, PhD

**Coordinator**: Henrique Disessa, MSc

**Collaborating researchers**: None.

**Centers (Juiz de Fora)**: 1) DaVita Juiz de Fora, Juiz de Fora, Minas Gerais

2) DaVita Rio Branco, Juiz de Fora, Minas Gerais

**Principal Investigator**: Maycon M. Reboredo, PhD

**Coordinator**: Marina Silveira, RD

**Collaborating researchers**: Emanuele P. L. Gravina, Ana C. C. Bainha

**Center**: Clínica de Nefrologia de Araranguá, Araranguá, Santa Catarina

**Principal Investigator**: Daiana Bundchen, PhD

**Coordinator**: Daiana Bundchen, PhD

**Collaborating researchers**: Christine Zomer Dal Molin, Camila Rocha Vignali, Beatriz Rocha Viana, Karine Pires Costa, Juliana dos Santos Raimundo, Laura Polo, Adriane Maria Horn, Lucas Alves Pizzutti, Gabrielli Vieira Carrer, Laís C. Carvalho, Josué dos Santos Barbosa Júnior, Barbara Marjorie Schwab, Rafaela Aguiar Rosa

**Center**: Fundação Pró-Rim, Joinville, Santa Catarina

**Principal Investigator**: Bruna M. Sant'Helena, PhD

**Coordinator**: Bruna M. Sant'Helena, PhD

**Collaborating researchers**: Rodolfo Nunes Bittencourt, Maria Cecília Kohler Panno

**Center**: Unidade de Terapia Renal Jorge Bandarra Westphalen do Hospital São Vicente de Paulo, Cruz Alta, Rio Grande do Sul

**Principal Investigator**: Rodrigo Krug, PhD

**Coordinator**: Rodrigo Krug, PhD

**Collaborating researchers**: Paulo Ricardo Moreira, PhD, Thais Severo Dutra, Jailton Possebom Marsola, Anny Beatriz Somavilla, Eduarda Martins Machado, Taiene Rodrigues, Amiria Teixeira Santana

**Center**: Unidade de Hemodiálise do Hospital de Clínicas de Porto Alegre (HCPA), Porto Alegre, Rio Grande do Sul

**Principal Investigator**: Angélica Adamoli, PhD

**Coordinator**: Angélica Adamoli, PhD

**Collaborating researchers**:  Catiussa Colling, Rodrigo Jacobsen, Sabrina Rodrigues da Silva, Deise dos Santos Farias, Júlia Rodrigues, Raíssa Teixeira

**Center**: Hospital Universitário São Francisco, Pelotas, Rio Grande do Sul

**Principal Investigator**: Maristela Bohlke, PhD

**Coordinator**: Maristela Bohlke, PhD

**Supplementary Table 1.** Diagnosis of electrolyte imbalances.

| **Electrolyte** | **Imbalance** | **Cutoff value** | **Reference** |
| --- | --- | --- | --- |
| Sodium (mEq/L) | Hyponatremia | < 135.0 | ^1^ |
| Potassium (mEq/L) | Hyperkalemia | > 5.0 | ^1^ |
| Phosphorus (mg/dL) | Hyperphosphatemia | > 4.5 | ^1^ |
| Calcium (mg/dL) | Hypocalcemia | < 8.5 | ^2^ |
| Albumin (g/dL) | Hypoalbuminemia | < 3.5 | ^3^ |
| iPTH (pg/mL) | High | > 600 | ^4*^ |
|  | Normal | 150 – 600 |  |
|  | Low | < 150 |  |

iPTH, intact parathyroid hormone.

* values were rounded.

1. National Kidney Foundation. Understanding your lab values and other CKD health numbers. *National Kidney Foundation*, https://www.kidney.org/atoz/content/understanding-your-lab-values.

2. Gudmundsdottir A, Doelle G. Hypercalcemia and Hypocalcemia. In: Bar RS (ed) *Early Diagnosis and Treatment of Endocrine Disorders*. Totowa, NJ: Humana Press, 2003, pp. 277–295.

3. Gatta A, Verardo A, Bolognesi M. Hypoalbuminemia. *Intern Emerg Med* 2012; 7: 193–199.

4. Stevens PE, Ahmed SB, Carrero JJ, et al. KDIGO 2024 Clinical Practice Guideline for the Evaluation and Management of Chronic Kidney Disease. *Kidney Int* 2024; 105: S117–S314.

**Supplementary Table 2.** Missing data and imputation.

|  | **Absolute (n)** | **Relative (%)** | **Imputation** |
| --- | --- | --- | --- |
| Sex | 0 | 0 | - |
| Age | 0 | 0 | - |
| Ethnicity | 1 | 0.1 | no |
| Marital status | 1 | 0.1 | no |
| Education level | 2 | 0.2 | no |
| Minimum wages | 98 | 10.0 | no |
| Smoking | 7 | 0.7 | no |
| Alcohol | 8 | 0.8 | no |
| **Comorbidities** |  |  |  |
| Diabetes | 2 | 0.2 | no |
| Hypertension | 2 | 0.2 | no |
| Cancer | 3 | 0.3 | no |
| COPD | 3 | 0.3 | no |
| Heart failure | 4 | 0.4 | no |
| CAD | 3 | 0.3 | no |
| Neuropathies | 5 | 0.5 | no |
| CKD etiology | 13 | 1.3 | no |
| Dialysis modality | 1 | 0.1 | no |
| Dialysis vintage (months) | 1 | 0.1 | yes |
| Weekly frequency | 0 | 0 | - |
| Vascular access | 0 | 0 | - |
| Weight | 10 | 1.0 | no |
| Height | 1 | 0.1 | no |
| **Laboratory parameters** |  |  |  |
| Sodium | 133 | 13.5 | yes |
| Potassium | 39 | 4.0 | yes |
| Phosphorus | 44 | 4.5 | yes |
| Calcium | 40 | 4.1 | yes |
| Albumin | 66 | 6.7 | yes |
| iPTH | 110 | 11.2 | yes |
| **Body composition** |  |  |  |
| Body mass index | 9 | 0.9 | yes |
| Calf circumference | 80 | 8.1 | no |
| Midarm muscle circumference | 87 | 8.9 | no |
| Tricipital skinfold | 86 | 8.7 | no |
| **Physical function** |  |  |  |
| Handgrip strength | 0 | 0 | - |
| Gait speed | 76 | 7.7 | no |
| Five-time sit-to-stand | 81 | 8.2 | no |
| Physical activity levels | 57 | 5.8 | yes |

CAD, coronary artery disease; COPD, chronic obstructive pulmonary disease; CKD, chronic kidney disease; iPTH, intact parathyroid hormone.

**Supplementary Table 3.** Sociodemographic and clinical characteristics of the patients on hemodialysis.

|  | **All patients** | **No sarcopenia** | **Probable sarcopenia** | **Confirmed sarcopenia** | **Severe sarcopenia** | ***p*-value** |
| --- | --- | --- | --- | --- | --- | --- |
| n (%) | 983 (100) | 729 (74.2) | 115 (11.7) | 88 (9.0) | 51 (5.2) |  |
| **Marital status**, n (%) |  |  |  |  |  | 0.075 |
| Married | 545 (55.5) | 403 (55.3) | 60 (52.2) | 52 (59.1) | 30 (60.0) |  |
| Divorced | 117 (11.9) | 88 (12.1) | 11 (9.6) | 13 (14.8) | 5 (10.0) |  |
| Widow | 107 (10.9) | 72 (9.9) | 21 (18.3) | 5 (5.7) | 9 (18.0) |  |
| Single | 213 (21.7) | 166 (22.8) | 23 (20.0) | 18 (20.5) | 6 (12.0) |  |
| **Education level**, n (%) |  |  |  |  |  | 0.688 |
| Illiterate | 36 (3.7) | 29 (4.0) | 3 (2.6) | 2 (2.3) | 2 (4.1) |  |
| Elementary | 358 (36.5) | 256 (35.1) | 48 (41.7) | 35 (39.8) | 19 (38.8) |  |
| Secondary | 335 (34.1) | 258 (35.4) | 30 (26.1) | 31 (35.2) | 16 (32.7) |  |
| Graduation | 207 (21.1) | 152 (20.9) | 30 (26.1) | 17 (19.3) | 8 (16.3) |  |
| Post-graduation | 45 (4.6) | 34 (4.7) | 4 (3.5) | 3 (3.4) | 4 (8.2) |  |
| **Minimum wages**, n (%) |  |  |  |  |  | 0.060 |
| 1 – 2 | 556 (63.0) | 418 (63.7) | 60 (60.0) | 56 (67.5) | 22 (51.2) |  |
| 3 – 4 | 141 (16.0) | 111 (16.9) | 16 (16.0) | 10 (12.0) | 4 (9.3) |  |
| ≥ 5 | 185 (21.0) | 127 (19.4) | 24 (24.0) | 17 (20.5) | 17 (39.5) |  |
| **Smoking**, n (%) |  |  |  |  |  | 0.491 |
| Yes | 71 (7.3) | 52 (7.2) | 5 (4.3) | 10 (11.4) | 4 (8.0) |  |
| No | 661 (67.7) | 483 (66.8) | 82 (71.3) | 61 (69.3) | 35 (70.0) |  |
| Never | 244 (25.0) | 188 (26.0) | 28 (24.3) | 17 (19.3) | 11 (22.0) |  |
| **Alcohol**, n (%) |  |  |  |  |  | 0.623 |
| Yes | 161 (16.5) | 124 (17.2) | 15 (13.0) | 13 (14.8) | 9 (18.0) |  |
| No | 712 (73.0) | 519 (71.9) | 86 (74.8) | 69 (78.4) | 38 (76.0) |  |
| Never | 102 (10.5) | 79 (10.9) | 14 (12.2) | 6 (6.8) | 3 (6.0) |  |
| **CKD etiology**, n (%) |  |  |  |  |  | 0.006 |
| Diabetes | 220 (22.7) | 144 (20.1) | 30 (26.3) | 30 (34.1) | 16 (32.0) |  |
| Hypertension | 260 (26.8) | 184 (25.6) | 34 (29.8) | 24 (27.3) | 18 (36.0) |  |
| Glomerulonephritis | 92 (9.5) | 80 (11.1) | 9 (7.9) | 2 (2.3) | 1 (2.0) |  |
| Polycystic kidneys | 65 (6.7) | 53 (7.4) | 5 (4.4) | 6 (6.8) | 1 (2.0) |  |
| Others | 164 (16.9) | 121 (16.8) | 19 (16.7) | 13 (14.8) | 11 (22.0) |  |
| Unknown | 165 (71.2) | 136 (18.9) | 17 (14.9) | 13 (14.8) | 3 (6.0) |  |
| **Serum markers**, mean |  |  |  |  |  |  |
| Sodium (mEq/L) | 138.5 ± 3.1 | 138.5 ± 3.1 | 138.5 ± 3.4 | 138.6 ± 3.1 | 137.6 ± 2.7 | 0.221 |
| Potassium (mEq/L) | 5.1 ± 0.8 | 5.1 ± 0.8 | 5.1 ± 0.8 | 5.0 ± 0.7 | 5.0 ± 0.8 | 0.965 |
| Phosphorus (mg/dL) | 5.2 ± 1.5 | 5.3 ± 1.6 | 5.0 ± 1.3 | 5.2 ± 1.5 | 4.5 ± 1.4^a^ | 0.001 |
| Calcium (mg/dL) | 8.8 ± 0.8 | 8.8 ± 0.8 | 8.7 ± 0.7 | 8.8 ± 0.7 | 8.9 ± 0.8 | 0.718 |
| Albumin (g/dL) | 3.9 ± 0.4 | 3.9 ± 0.4 | 3.9 ± 0.4 | 3.8 ± 0.4 | 3.7 ± 0.4^a^ | 0.007 |
| iPTH (pg/mL)***** | 345 [177 – 643] | 359 [187 – 669] | 329 [197 – 503] | 355 [154 – 593] | 236 [124 – 495] | 0.067 |
| **Dialysis weekly frequency**, n (%) | | |  |  |  | 0.007 |
| Two | 18 (1.8) | 9 (1.2) | 3 (2.6) | 3 (3.4) | 3 (5.9) |  |
| Three | 677 (68.9) | 509 (69.8) | 83 (72.2) | 60 (68.2) | 25 (49.0) |  |
| Four | 88 (9.0) | 73 (10.0) | 5 (4.3) | 6 (6.8) | 4 (7.8) |  |
| Five | 104 (10.6) | 70 (9.6) | 11 (9.6) | 10 (11.4) | 13 (25.5) |  |
| Six | 96 (9.8) | 69 (9.3) | 13 (11.3) | 9 (10.2) | 6 (11.8) |  |

CKD, chronic kidney disease; iPTH, intact parathyroid hormone.

* reported in median and interquartile range.

^a^, ^b^, and ^c^ indicate significant differences to the no sarcopenia, probable sarcopenia, and confirmed sarcopenia groups, respectively.

**Supplementary Table 4**. Sensitivity analyses for the factors independently associated with sarcopenia in patients on hemodialysis.

| **Variables** | **Adjusted model** | |
| --- | --- | --- |
|  | **Odds ratio (95% CI)** | ***p*-value** |
| **Excluding patients on hemodiafiltration** |  |  |
| Older age (≥ 60 years) | 2.46 (1.41 – 4.26) | 0.001 |
| Male sex | 1.35 (0.77 – 2.34) | 0.013 |
| White ethnicity | 2.24 (1.31 – 3.85) | 0.003 |
| Diabetes as CKD etiology or comorbidity | 1.63 (0.96 – 2.77) | 0.071 |
| Body mass index |  |  |
| Normal weight | **reference** | |
| Underweight | 5.67 (0.53 – 60.58) | 0.144 |
| Overweight | 0.37 (0.21 – 0.66) | <0.001 |
| Obesity | 0.09 (0.03 – 0.29) | <0.001 |
| **Excluding patients on short daily dialysis frequency (≥ 4 sessions/week)** | |  |
| Older age (≥ 60 years) | 2.81 (1.64 – 4.84) | <0.001 |
| Male sex | 1.38 (0.81 – 2.35) | 0.238 |
| White ethnicity | 2.13 (1.27 – 3.56) | 0.004 |
| Diabetes as CKD etiology or comorbidity | 1.54 (0.92 – 2.57) | 0.100 |
| Body mass index |  |  |
| Normal weight | **reference** | |
| Underweight | 5.96 (0.56 – 63.96) | 0.140 |
| Overweight | 0.39 (0.22 – 0.68) | <0.001 |
| Obesity | 0.09 (0.03 – 0.29) | <0.001 |
| Hemodiafiltration | 1.42 (0.73 – 2.74) | 0.301 |

CKD, chronic kidney disease; CI, confidence interval.

Confirmed and severe sarcopenia groups were considered as ‘sarcopenia’ for analysis.

Adjusted model included age (reference = <60 years), ethnicity (reference = non-white), sex (reference = female), diabetes as etiology or comorbidity (reference = no diabetes), body mass index (reference = normal weight), dialysis modality (reference = conventional) and vascular access (reference = arteriovenous fistula or graft).

Dialysis modality was not included in the sensitivity analysis for hemodiafiltration.


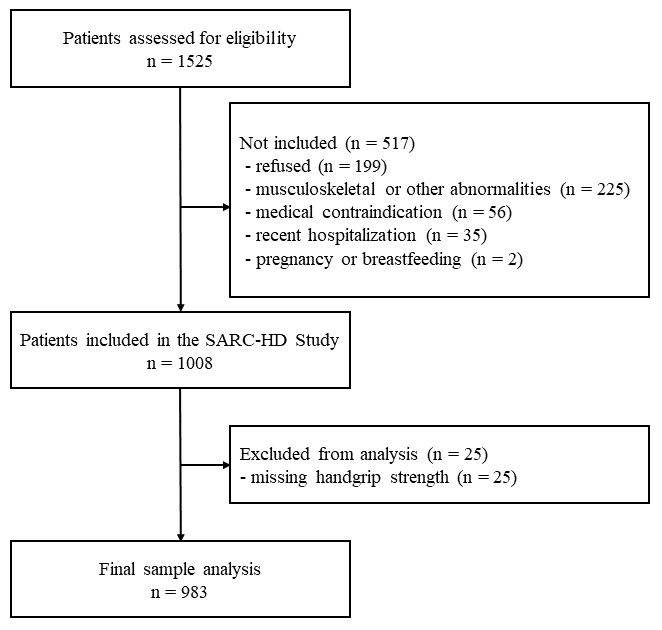


**Supplementary Figure 1**. Recruitment flowchart of the SARC-HD study.


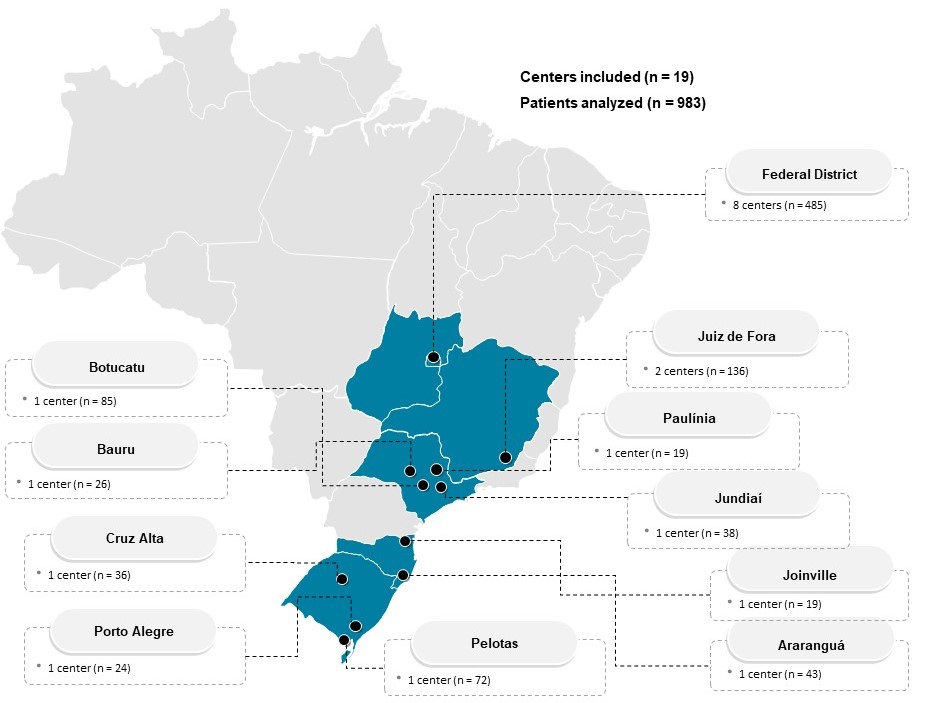


**Supplementary Figure 2**. Map of Brazil with the distribution of centers and patients included in the SARC-HD study.


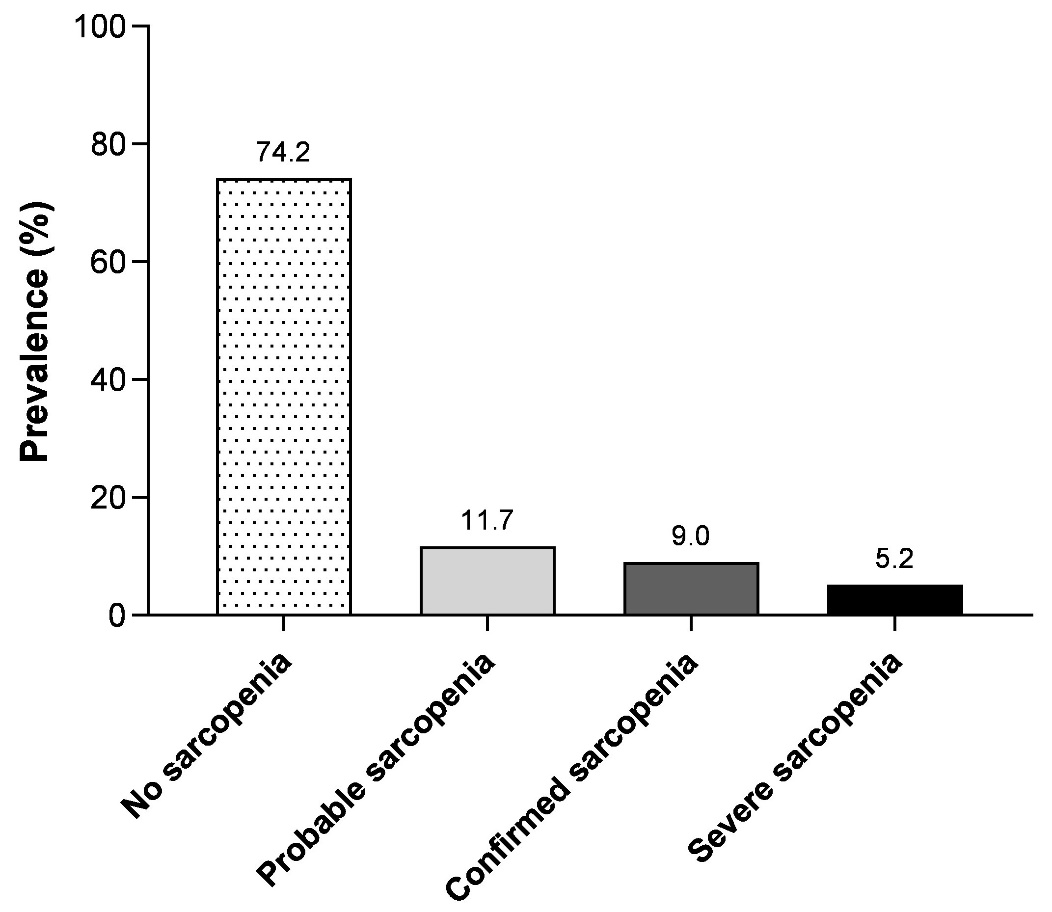


**Supplementary Figure 3**. Prevalence of sarcopenia stages.

**
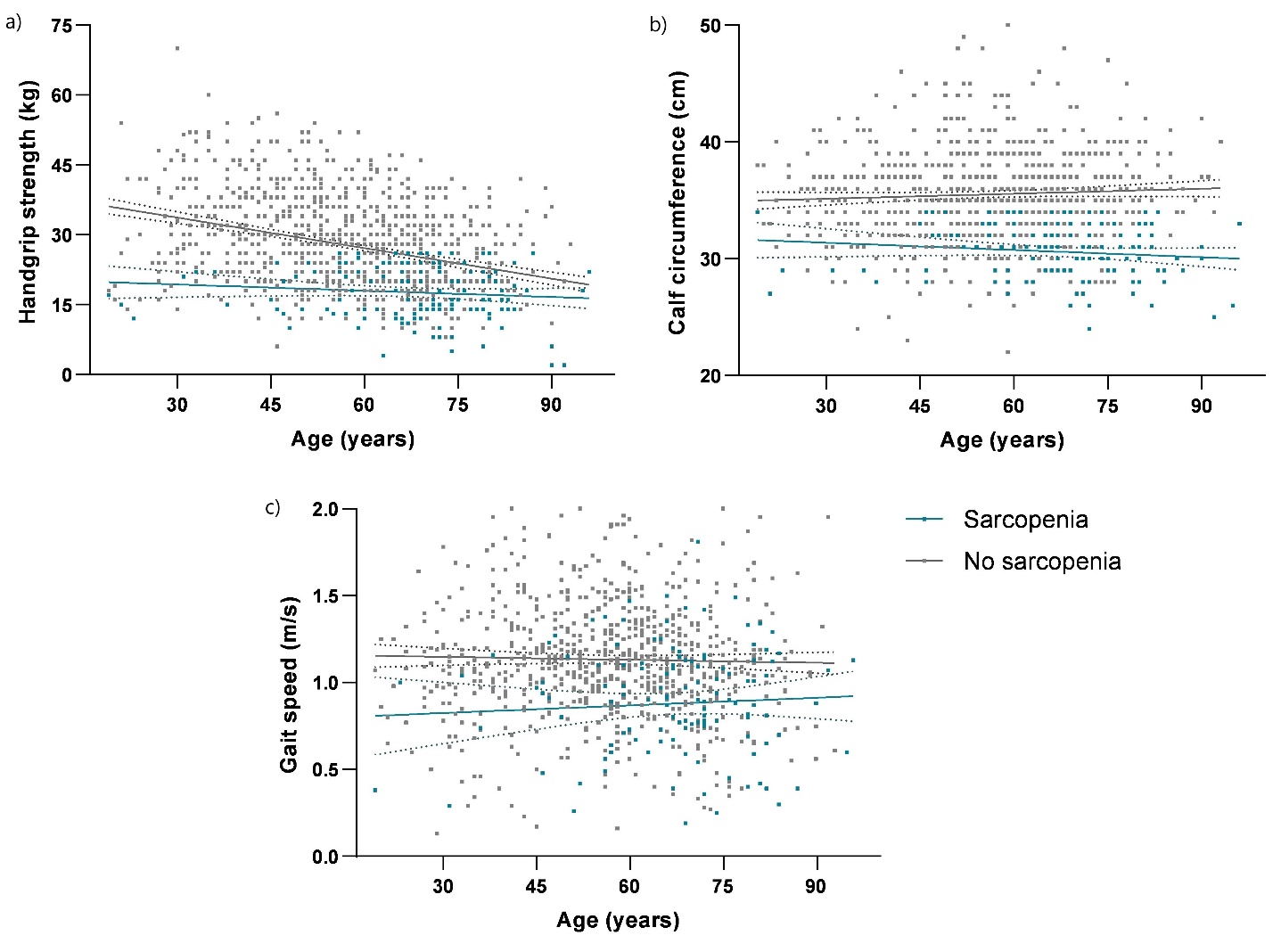
Supplementary Figure 4.** Association between age and sarcopenia-related variables stratified by sarcopenia status.

a) handgrip strength; b) calf circumference; c) gait speed.

Sarcopenia was defined as the combination of the confirmed and severe stages.
